# A Backpack’s Worth of Data: Elevated Teen Cancer Risks Linked to Air Pollution

**Published:** 2006-10

**Authors:** M. Nathaniel Mead

It is difficult to assess the cancer risks associated with exposures to air pollutants because much of the health focus has been on the major outdoor pollutants; far less is known about exposures inside homes and buildings, where pollutants may be far more concentrated. Better assessments would measure personal exposures to inhaled pollutants, but such measurements are both costly and challenging to collect. Now a research team has developed an effective method to monitor personal exposures to air pollution with the goal of estimating cancer risks for indoor and outdoor exposures in urban areas **[*EHP* 114:1558–1566; Sax et al.]**.

The team recruited 87 high school students from Los Angeles and New York City. Three types of measurements were obtained: indoor home samples, outdoor home samples, and personal exposure samples. To obtain personal exposure samples, each teenager wore a regular backpack modified to carry sampling equipment and various types of samplers for aldehydes, particles, and volatile organic compounds (VOCs). This enabled sampling of air wherever the teenager spent time over a 48-hour period, providing an integrated measurement of the air exposures from all indoor and outdoor environments. The personal, ambient, and modeled concentrations were used together with EPA data and other toxicological information to determine excess cancer risks associated with the exposure levels.

In both cities, median cancer risks from personal VOC exposures were much greater than from ambient exposures. Of the VOCs measured, formaldehyde carried the greatest cancer risk (more than 1 in 1 million, based on current EPA data), despite the decline of indoor levels since the banning of formaldehyde foam insulation. Levels of 1,4-dichlorobenzene also posed a substantial risk, though the carcinogenicity of the compound is uncertain, and the risk applied only to teenagers who had sources of this compound (e.g., toilet bowl deodorizers) in their homes. Benzene, the only known human carcinogen in the group of VOCs, posed the greatest risk from outdoor sources. Automobile exhaust and tobacco smoke are two major sources of this pollutant.

This study is unique in its focus on teenage groups in two of the nation’s largest and most polluted cities. The two cities have different climates and different housing and commuting options, all factors that can influence exposures to both outdoor and indoor pollutants. Surprisingly, however, the two cities differed little in terms of overall cancer risks. In both cities, indoor exposures were a large determinant of cancer risks.

It is unclear whether the results of this study can be generalized to teens living in other urban areas. Large cancer risks were associated with exposures to many of the VOCs studied, but the toxicological data used to determine these risks have substantial uncertainty. Still, the findings provide valuable assessments of the risks associated with inhaling a suite of pollutants, both gaseous and particle-bound, based on personal exposure measurements that can be used to improve modeled estimates.

## Figures and Tables

**Figure:**
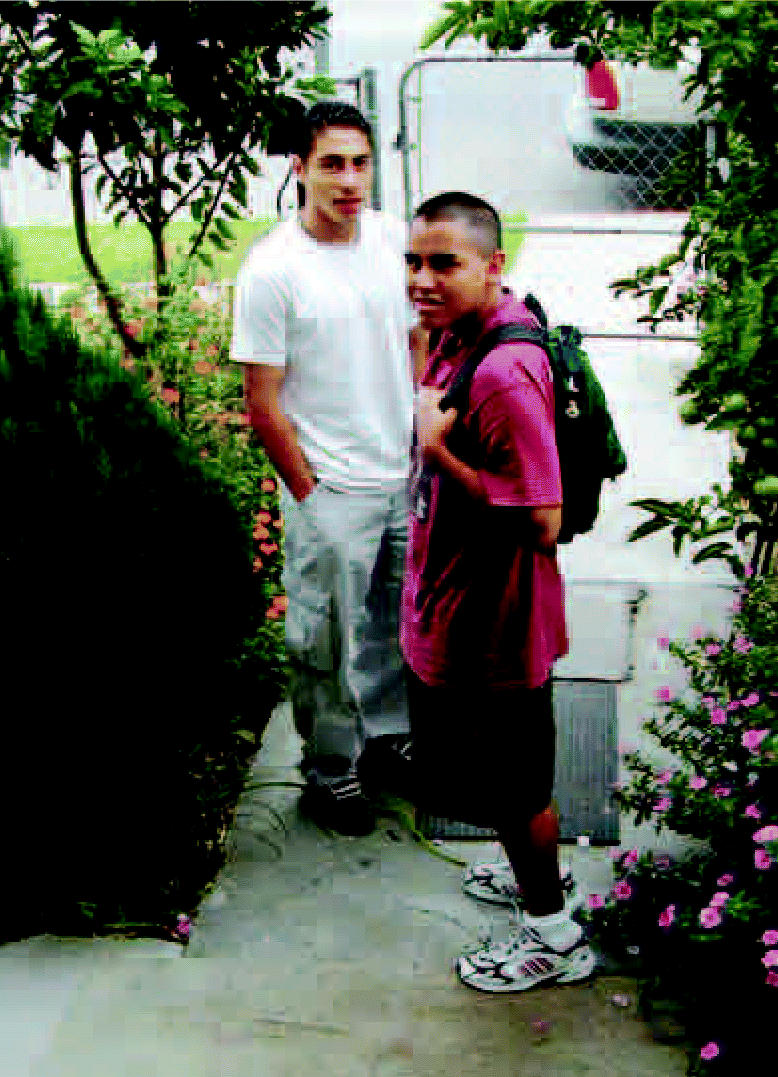
It goes where they go A new backpack monitor records teens’ personal exposures to pollutants.

